# Monitoring reversion of hepatitis C virus-induced cellular alterations by direct-acting antivirals using cryo soft X-ray tomography and infrared microscopy

**DOI:** 10.1107/S2059798321009955

**Published:** 2021-10-27

**Authors:** Ana J. Perez-Berna, Nuria Benseny-Cases, María José Rodríguez, Ricardo Valcarcel, José L. Carrascosa, Pablo Gastaminza, Eva Pereiro

**Affiliations:** a ALBA Synchrotron Light Source, Carrer de la Llum 2–26, 08290 Cerdanyola del Valles, Spain; b Centro Nacional de Biotecnología, Campus de Cantoblanco, Universidad Autónoma de Madrid, Madrid, Spain

**Keywords:** cryo soft X-ray tomography, hepatitis C virus, direct-acting antivirals, synchrotron-based infrared microscopy, membranous web

## Abstract

Hepatitis C virus (HCV) is an enveloped RNA virus. One of the hallmarks of HCV infection is a rearrangement of the host cell membranes, known as the ‘membranous web’. Full-field cryo soft X-ray tomography (cryo-SXT) in the water-window energy range (284–543 eV) was performed on the MISTRAL beamline to investigate, in whole unstained cells, the morphology of the membranous rearrangements induced in HCV replicon-harbouring cells in conditions close to the living physiological state. All morphological alterations could be reverted by a combination of sofosbuvir/daclatasvir, which are clinically approved antivirals (direct-acting antivirals; DAAs) for HCV infection. Correlatively combining cryo-SXT and 2D synchrotron-based infrared microscopy provides critical information on the chemical nature of specific infection-related structures, which allows specific patterns of the infection process or the DAA-mediated healing process to be distinguished.

## Abstract 2

The study of cells replicating hepatitis C and treated with antiviral compounds by soft X-ray cryo tomography (cryo-SXT) and synchrotron-based infrared microscopy allows correlation of the viral structures resolved by the former with their chemical composition revealed by the latter. The results show the potential of cryo-SXT as a platform to determine the effectiveness of antiviral compounds in infected cells, guiding drug development at a preclinical level.

## 1. Introduction   

The prevalence of chronic hepatitis C virus (HCV) infection has been estimated at 1.2–1.7% of the adult global population. Until recently, HCV infection was treated using interferon alone or in combination with different drugs, giving low rates of sustained virological response (SVR) accompanied by a number of adverse effects. A paradigm shift occurred in late 2014 with the approval of interferon-free direct-acting antiviral (DAA) therapeutic regimens using drug combinations that target various steps of the HCV genome-replication cycle by directly binding to components of the replicase complex or by causing RNA chain termination. Since then, new drug combinations have led to SVR rates of above 95% in all treated patients, both in clinical trials and in real-life clinical practice. However, the limited availability of these treatments as well as cost considerations are still important limitations to implementing these treatments globally (Kish *et al.*, 2017 ▸).

HCV is a member of the *Flaviviridae* family of enveloped RNA viruses. The HCV genome consists of a single-stranded RNA molecule of positive polarity that encodes a unique open reading frame, which is flanked by untranslated RNA regions that regulate virus translation and replication. The HCV polyprotein is processed by viral and cellular proteases to produce ten major proteins. Core, E1 and E2 are structural components of the virion, and the nonstructural proteins NS3, NS4A, NS4B, NS5A and NS5B are sufficient to support HCV RNA replication. The viral proteins p7 and NS2 coordinate the encapsidation of progeny genomes into infectious virions in coordination with the structural proteins and elements of the viral replicase (Moradpour & Penin, 2013 ▸). The HCV lifecycle is initiated by the entry of infectious virions into target cells by a multistep mechanism. Viral entry involves a number of cellular coreceptors that intervene through clathrin-mediated endocytosis and low-pH-mediated membrane fusion, resulting in the release of the viral genome into the cytoplasm (Asselah *et al.*, 2010 ▸). Once the polyprotein has been translated and processed into individual proteins, viral replicase complexes are assembled, generating progeny genomes using a full-length, negative-strand intermediate as a template. These progeny genomes are either translated or encapsidated into infectious virions that are assembled and secreted by mechanisms involving elements of the very low-density lipoprotein biosynthetic machinery (Asselah *et al.*, 2010 ▸). One of the hallmarks of HCV infection is a rearrange­ment of the host cell membranes, known as the membranous web. This membranous web is likely to host the viral replicase, as viral RNA and nonstructural proteins have been localized within these vesicular structures (Romero-Brey *et al.*, 2012 ▸; Romero-Brey & Bartenschlager, 2014 ▸). HCV-infected cells show vesicular alterations that can be classified as single- and double-membrane vesicles, and multiple-membrane vesicles. 3D reconstruction of HCV-infected cell sections revealed that the membranous web, which is likely to be derived from modified endoplasmic reticulum cisternae, consists of enlarged endoplasmic reticulum tubules with double-membrane vesicles that are either connected or tightly apposed to the endoplasmic reticulum, with a minor proportion of double-membrane vesicles separated by the endoplasmic reticulum network (Romero-Brey *et al.*, 2012 ▸; Romero-Brey & Bartenschlager, 2014 ▸). Subsequent biochemical studies support the notion that the double-membrane vesicles contain functional RNA replicase complexes, similarly to as has been proposed for other positive-strand RNA viruses (Romero-Brey & Bartenschlager, 2014 ▸). In previous work, we obtained complete 3D structures of control cells and cells harbouring HCV replicons in the native state by cryo soft X-ray tomography (cryo-SXT) on the MISTRAL beamline at the ALBA Synchrotron (Pérez-Berná *et al.*, 2016 ▸). Cryo-SXT is the only imaging modality that allows the collection of 3D maps of regions of whole vitrified cells at nanometre resolution, thus avoiding chemical treatment or sectioning, by taking advantage of their natural contrast in the water-window energy range (between the *K* absorption edges of C and O; Sorrentino *et al.*, 2015 ▸; Pereiro *et al.*, 2009 ▸) with an achievable half-pitch resolution of down to 28 nm (Reineck *et al.*, 2021 ▸; Otón *et al.*, 2016 ▸). We have previously identified the salient alterations induced by HCV replication as well as the sequence of early events occurring in a surrogate model of HCV infection (Pérez-Berná *et al.*, 2016 ▸). In addition, we also studied the regression of the pathological membranous web proliferation by the use of the experimental inhibitor 2-*C*-methyladenosine (2MAd) and clinically relevant DAAs (Pawlotsky, 2013 ▸) such as daclatasvir, telaprevir and sofosbuvir, demonstrating that the observed alterations were indeed induced by HCV replication and reverted after seven and 20 days of treatment (Pérez-Berná *et al.*, 2016 ▸).

Here, we establish a spatial correlation between the ultrastructural changes occurring in HCV-replicating cells and the protein and lipid alterations induced by viral replication using correlative cryo-SXT and synchrotron-based infrared microscopy (SR-μFTIR). The correlated data permit the establishment of a relationship between the membranous web in the cells and the specific chemical footprint of lipids and proteins. Note that the membranous web spreads over large cytoplasm regions, which are usually larger than the field of view of the tomograms collected, while SR-μFTIR microscopy allows the scanning of whole cells, although at a resolution of 8 µm. Stitching of tomograms to cover larger regions is possible provided that the total dose is within the damage limit.

SR-μFTIR shows important alterations in lipid oxidation and a remarkable increase in the lipid:protein absorbance ratio, as well as alterations in protein folding across the cytoplasm of HCV-replicating cells, with a significant increase in β-sheet protein conformation. In order to integrate all of the data into meaningful models, we used principal component analysis (PCA), a multivariate statistical analysis that is applied here to SR-μFTIR data. This method, which is based on comparison of the covariance spectra of the whole data set, allows classification of the spectra depending on their similarities and differences, and is used for pathological and biomedical investigation of different types of cells and tissues. PCA analysis reveals specific spectroscopic signatures in HCV-replicating cells compared with controls. Moreover, analyses of DAA-cured cells reveal FTIR spectra similar to those of control cells.

Our results constitute a proof of concept for the use of cryo-SXT and SR-μFTIR as a platform that enables the determination of the potential impact of candidate compounds on the cell ultrastructure and chemical composition, which may assist drug development at a preclinical level.

## 2. Methods   

### 2.1. Cell cultures   

Human hepatoma cell line HuH-7 was cultured as described previously in Dulbecco’s modified Eagle’s medium supplemented with HEPES, nonessential amino acids, penicillin/streptomycin and 10% fetal calf serum (complete medium; Zhong *et al.*, 2005 ▸). Replicon cell lines were produced as described previously (Kato *et al.*, 2003 ▸). Briefly, HuH-7 cells were electroporated with an *in vitro* transcribed genotype 2a subgenomic dicistronic HCV RNA from the JFH-1 strain. The first cistron encodes the *neo* (neomycin phosphotransferase) gene that confers resistance to the antibiotic G418 and is translated under the control of the HCV 5′-UTR (untranslated region). The second cistron encodes the genomic region of the HCV genome encoding the viral replicase components from NS3 to NS5B (NS3, NS4A, NS4B, NS5A and NS5B) under the translational control of EMCV IRES and the HCV genome 3′-UTR. Effective replication of this RNA genome confers resistance to G418 selection to the host cell. Thus, transfected cells were subjected to 250 µg ml^−1^ G418 treatment until the control, mock-transfected cell population had disappeared. The resistant cell population was analyzed by Western blotting using antibodies against NS3 from Biofront Tech (Tallahassee, Florida, USA) and against human β-actin as a loading control (ab8226; Abcam, Cambridge, UK), and by RT-qPCR to quantify the viral RNA load on total cellular RNA as described previously (Friesland *et al.*, 2013 ▸; Zhong *et al.*, 2005 ▸). Aliquots of these cells were cryopreserved in liquid nitrogen and used throughout the different experiments.

In the inhibitor-treatment experiments, replicon cells were treated with either 10 µ*M* 2-*C*-methyladenosine (2MAd), daclatasvir (Dac; 1 n*M*), sofosbuvir (Sof; 1 µ*M*) or a combination of Dac (1 n*M*) and Sof (1 µ*M*) for seven and 20 days of treatment, replacing the cultures with fresh media and drugs when split. The effectiveness of the antiviral treatment was assessed at days 7 and 20 by Western blotting against NS3 and RT-qPCR as described above. The samples used in this study are identical to those reported in Pérez-Berná *et al.* (2016 ▸).

### 2.2. Soft X-ray cryo-tomography   

Adherent cells were trypsinized, resuspended at a concentration of 5 × 10^5^ cells ml^−1^ and seeded onto gold Quantifoil R 2/2 holey carbon-film microscopy grids (Au-G200F1); 24 h later they were vitrified by plunge-freezing in a Leica EMCPC. Before vitrification, fiducial gold markers (100 nm; BBI Solutions, UK) were added for tomographic alignment purposes. The frozen grids were imaged using a Linkam CMS196 stage in a Zeiss Axioscope fluorescence microscope and were then transferred to the MISTRAL beamline (Pereiro *et al.*, 2009 ▸; Sorrentino *et al.*, 2015 ▸) at the ALBA Synchrotron under cryogenic conditions. We used a photon energy (520 eV) within the water window to take advantage of the high natural absorption contrast of the biological material to acquire X-ray tomography data sets using the conditions described above in Section 2.1 (Pérez-Berná *et al.*, 2016 ▸). The data sets were acquired using a Fresnel zone-plate objective lens with an outermost zone width of 40 nm. The effective pixel size was 9.8 nm. The projection stacks were normalized and corrected by the machine current, as well as deconvolved by the measured point-spread function (PSF; Otón *et al.*, 2016 ▸). Alignment of the projections and erasure of the gold fiducials of the tilt series was performed with *IMOD* (Kremer *et al.*, 1996 ▸). The final reconstructions were performed using the iterative SIRT reconstruction option in *TOMO*3*D* (Agulleiro & Fernandez, 2011 ▸). To enhance the signal-to-noise levels, we used *TOMOEED* (Fernández & Li, 2003 ▸). The visualization and segmentation of the final volumes were carried out in the *Amira-Avizo* software and *UCSF Chimera* (Pettersen *et al.*, 2004 ▸). The 3D resolution estimation was performed using the same approach as described previously in Carrascosa *et al.* (2009 ▸).

### 2.3. Fixation   

After cryo-SXT, the Quantifoil cryofixed grids were dipped from liquid nitrogen into a 3.7% paraformaldehyde (PFA) solution at room temperature for 30 min and then dipped three times into double-distilled water. The cells were dried in a desiccator for at least 12 h before SR-μFTIR acquisition. The adequateness of the fixation procedure using PFA and its lack of contribution to the spectra is reported in Mazur *et al.* (2012 ▸).

### 2.4. Synchrotron-based Fourier transform infrared microspectroscopy (SR-μFTIR) and data acquisition   

The same cells as analysed by SXT after PFA fixation were measured by SR-μFTIR on the MIRAS beamline at the ALBA Synchrotron using a Hyperion 3000 Microscope equipped with a 36× magnification objective coupled to a Vertex 70 spectrometer (Bruker) (Yousef *et al.*, 2017 ▸). The measuring range was 650–4000 cm^−1^. Spectra collection was carried out in transmission mode at 4 cm^−1^ resolution with aperture dimensions of 8 × 8 µm and from 128 to 256 scans. Zero filling was performed with the fast Fourier transform so that in the final spectra there was one point every 2 cm^−1^. Background spectra were collected from an empty area (a region without Quantifoil) every 15 min. An MCT detector with 50 µm resolution, a responsivity at 4.8 mA of 173 550 V W^−1^ and a field of view (FOV) of 45 µm was used. The microscope and spectrometer were continuously purged with nitrogen gas.

### 2.5. Analysis of FTIR spectra   

Spectra were acquired in two different ways: (i) at least 50 spectra from single cells were acquired for each sample in a given sample region and (ii) maps with minimum dimensions of 50 × 50 µm and a step size of 6 × 6 µm were collected. FTIR spectra of single independent cells and the spectra from the different cell maps were analyzed using *Opus* 7.5 (Bruker). Spectra exhibiting a low signal-to-noise ratio were eliminated. Resonant Mie scattering (RMieS) correction was carried out using the software freely provided online by Peter Gardner’s laboratory at the University of Manchester (Bassan *et al.*, 2012 ▸; Bassan, Kohler, Martens, Lee, Jackson *et al.*, 2010 ▸; Bassan, Kohler, Martens, Lee, Byrne *et al.*, 2010 ▸), implemented in *MATLAB*, involving ten iterations in the range 3100–1300 cm^−1^ using a scattering-particle diameter from 2 to 8 µm. The effect of the RMieS correction was performed on the whole set of spectra (a total of 3231 spectra). For data processing, the second derivative of the spectra was calculated using a Savitsky–Golay algorithm with a 13-point filter and a polynomial order of 2 to eliminate the baseline contribution. Derivatives were applied using 13 points. *Unscrambler X* (CAMO Software, Oslo, Norway) was used to perform PCA in the data set. PCA was applied to the second derivative of the spectra. Unit vector normalization was applied after secondary derivation for PCA (Baker *et al.*, 2014 ▸; Sandt *et al.*, 2016 ▸). Principal components (PCs) were calculated. Since the PCA procedure allows weighting of the individual variables relative to each other, a constant value (1.00 equal weight) was assigned to all variables (for different wavenumbers in the 650–4000 cm^−1^ region) as the recommended value. For PCA all RMieS corrected spectra (single-point spectra and the spectra from maps) were used. Absorbance ratios were calculated over the curve fitting of the following peaks of interest: 1635 cm^−1^ for amide I β-sheet structures (noted as *A*_1635_), 1656 cm^−1^ for α-helices (noted as *A*_1656_), 1740 cm^−1^ for ν(C=O) (carbonyl) (noted as *A*_1740_), 2925 cm^−1^ for CH_2_ asymmetric stretching vibrations (noted as *A*_2925_) and 2960 cm^−1^ for CH_3_ asymmetric stretching vibrations (noted as *A*_2960_) (Petibois *et al.*, 2007 ▸; Chwiej *et al.*, 2010 ▸; Benseny-Cases *et al.*, 2018 ▸). *Origin* 9.1 was used to calculate the ratios and to obtain graphical representations.

## 3. Results   

### 3.1. Correlative studies by cryo-SXT and SR-μFTIR of control and replicon-bearing human hepatoma cell lines   

**Figure 1:**
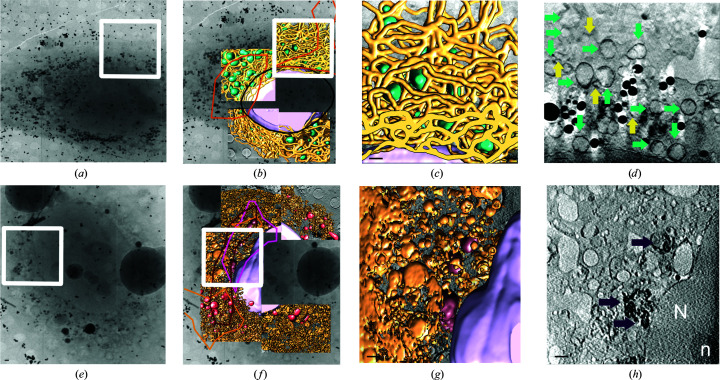
Three-dimensional reconstruction of whole-cell volumes of HCV replicon-bearing cell lines by cryo-SXT. Control (*a*–*d*) and HCV replicon-bearing (*e*–*h*) cell lines were vitrified and subjected to cryo-SXT. (*a*, *b*, *e*, *f*) Tile-scanned projections showing the area selected for SXT (white square); volume slices of the tomograms from the boxed areas (*c*, *d*, *g*, *h*) are also shown. Threshold-based isosurface segmentation of the surface boundaries identifies the different organelles present in the cells: normal mitochondria in green, abnormal mitochondria type I in red, abnormal mitochondria type II in purple, ER in yellow, modified ER in brown and nuclear envelope in pink. The same area of volume slice is shown in (*d*) and (*h*). Green and yellow arrows in (*d*) point out the normal mitochondria and ER, respectively. The abnormal mitochondria are pointed out by purple arrows in (*h*). The scale bars represent 1 µm.

**Figure 2:**
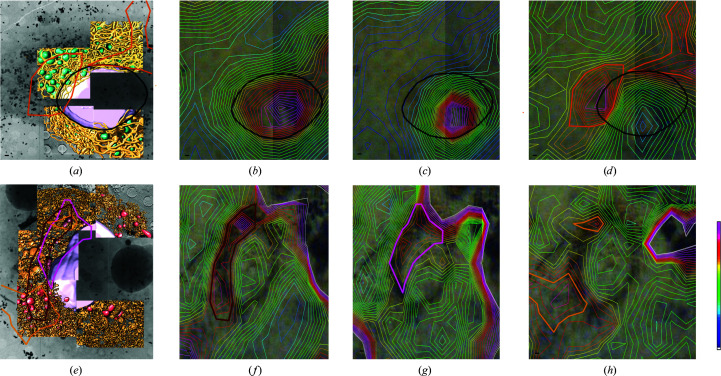
Correlative cryo-SXT and representative infrared and visible maps of control cells and HCV-replicating cells. (*a*, *e*) Three-dimensional reconstruction of whole-cell volumes of control and HCV replicon-bearing cell lines by cryo-SXT. Tile-scanned projections showing the area selected for SXT as well as volume tomograms are shown. A colour-coded manual segmentation of the surface boundaries identifying different organelles is presented. The normal mitochondria are segmented in green, abnormal mitochondria type I are segmented in red, abnormal mitochondria type II are segmented in purple, ER is segmented in yellow, modified ER is segmented in brown and nuclear envelope is segmented in pink. (*b*, *f*) Lipid area distribution, (*c*. *g*) Amide I area distribution. (*d*, *h*) Absorbance ratios corresponding to oxidation (*A*_1740_/*A*_2960+2925_). Blue is low intensity, red is high intensity. The scale bar represents 1 µm

We have analysed the ultrastructural and chemical alterations that liver cells undergo after HCV infection by cryo-SXT and SR-μFTIR. To comply with the biosafety protocols at the ALBA facility, we used a surrogate model of infection that did not involve the handling of infectious material. Thus, human hepatoma cell lines (HuH-7) bearing a subgenomic HCV replicon of genotype 2a (JFH-1 strain) were cryopreserved unstained and subjected to cryo-SXT on the MISTRAL beamline (Figs. 1 ▸, 2 ▸ *a* and 2 ▸ *e*). After these first experiments, the samples were recovered under cryogenic conditions and fixed by PFA for analysis by SR-μFTIR on the MIRAS beamline (Figs. 2 ▸ *b*, 2 ▸ *c*, 2 ▸ *d*, 2 ▸ *f*, 2 ▸ *g* and 2 ▸ *h*). Using our previous knowledge of the appearance of cellular organelles from cryo-STX (Pérez-Berná *et al.*, 2016 ▸), we were able to identify different subcellular compartments segmented in Figs. 1 ▸(*b*), 1 ▸(*c*), 1 ▸(*f*), 1 ▸(*g*), 2 ▸(*a*) and 2 ▸(*e*). Both in control and HCV-replicating cell nuclei, granular structures corresponding to chromatin fibres as well as the nucleoli can be identified (segmented in pink in Figs. 1 ▸ *b*, 1 ▸ *c*, 1 ▸ *f*, 1 ▸ *g*, 2 ▸ *a* and 2 ▸ *e* and labelled as N and n, respectively, in Fig. 1 ▸ *h*). No structural differences between the nuclei are found, indicating that HCV replication does not trigger any visible structural change in the nucleus, as expected.

Cryo-SXT of control cells revealed the presence of other structures, including organelles such as mitochondria (Figs. 1 ▸ *b* and 1 ▸ *c*; identified with a green arrow in Fig. 1 ▸ *d* and segmented in green in Figs. 1 ▸ *c* and 2 ▸ *a*), lipid droplets (black dots in Fig. 1 ▸ *d*) and endoplasmic reticulum (ER; Figs. 1 ▸ and 2 ▸ *a*; segmented in yellow and identified with a yellow arrow in Fig. 1 ▸ *d*), which appears as a membranous tubular structure with continuous cisternae and as tubular structures surrounding mitochondria (Figs. 1 ▸ *b*, 1 ▸ *c* and 2 ▸ *a*). It is important to remark that the ER is the largest organelle in the cell, consisting of two lipid bilayers with an intervening lumen that may vary in size and is usually about 50 nm. This size is difficult to observe in a reconstructed slice. In order to obtain an *in situ* representation of the chemical composition of the control cell, representative maps of the CH region, corresponding to the cellular lipid distribution, the amide I region and the *A*_1740_/*A*_2960+2925_ ratio, related to an increase in the number of carbonyl groups formed as a consequence of cell lipid oxidation, are shown in Figs. 2 ▸(*b*), 2 ▸(*c*) and 2 ▸(*d*), respectively. As expected, the nuclear region, delimited by a black line in the 2D maps of the control cell, clearly presents the highest area distribution of lipids and proteins (Figs. 2 ▸ *b* and 2 ▸ *c*), while specific regions of the cytoplasm show the highest values of the *A*_1740_/*A*_2960+2925_ ratio, which is related to areas with high numbers of carbonyl groups. In the 3D volumes generated by cryo-SXT, we were able to identify this cytoplasmic area as the region with the highest active mitochondria population in the cell, as delimited by an orange line in the segmented 3D volume (Fig. 2 ▸ *a*).

In contrast, the cytoplasm of the HCV-replicating cells displayed different heterogeneous vesicle-like structures in an intricate network of vesicular and tubular structures that are likely to be derived from the endoplasmic reticulum (Figs. 1 ▸ *f*, 1 ▸ *g* and 2 ▸ *e*), segmented in brown. These special membranous compartments have been shown to host viral replicase components, and are likely to be the site of HCV RNA replication (Romero-Brey & Bartenschlager, 2015 ▸). The membranous web compartment in HCV-replicating cells constitutes the most prominent virus-induced alteration in the cellular architecture (Mottola *et al.*, 2002 ▸). Another outstanding differential feature of the replicon cells is the aberrant mitochondrial morphology. This is in agreement with our previous reports indicating that HCV replication causes mitochondrial dysfunction. As has prevously been reported (Pérez-Berná *et al.*, 2016 ▸), abnormal mitochondria are characterized by increased absorption contrast, suggesting matrix condensation, as well as alterations in the diameter and the number of visible cristae compared with normal mitochondria (Figs. 1 ▸ *f*, 1 ▸ *g* and 1 ▸ *h*). Based on these criteria, abnormal mitochondria could be classified into two different morphological classes: class I (AbMito1) abnormal mitochondria, segmented in red, display a moderate increase in absorption contrast that is comparable to the surrounding cytosol, while class II (AbMito2), segmented in purple, clearly present a higher absorption contrast and the virtual loss of mitochondrial cristae (Pérez-Berná *et al.*, 2016 ▸; Figs. 1 ▸ *f* and 1 ▸ *g* and purple arrows in Fig. 1 ▸ *h*). The alterations observed in AbMito1 and AbMito2 constitute morphological hallmarks of mitochondrial dysfunction (Figs. 1 ▸ *f*, 1 ▸ *g*, 1 ▸ *h* and 2 ▸ *e*).

This 3D total reorganization of the HCV-replicating cells revealed clear chemical changes in the SR-μFTIR 2D maps, presenting an increase of lipid peaks in the perinuclear region (Fig. 2 ▸ *f*), delimited in the 3D volume by a dark red line, with the impact of membranous web expansion on the overall ultrastructure of the HCV-replicating cells (Fig. 2 ▸ *e*). Again, by correlating the SR-μFTIR 2D maps with the cryo-SXT cellular volumes, we can associate the maximum protein distribution (Fig. 2 ▸ *g*), delimited by a pink line, with the region where the mitochondria present the morphological dysfunction hallmarks corresponding to abnormal mitochondria (AbMito2), segmented in purple (Fig. 2 ▸ *e*). These areas display a strong membranous alteration of the virus-induced tubular network and correspond to a high oxidation parameter (*A*_1740_/*A*_2960+2925_ ratio; Fig. 1 ▸ *h*). This high oxidation ratio corresponds to the orange-delimited area in the 3D volume (Fig. 1 ▸ *e*). In this large cellular area, viral replication has induced a strong ER modification with ER enlargement and large tubular structures, producing the formation of the membranous web. Note that inside this membranous web the mitochondria present important morphological modifications that we have classified as class II (AbMito2) and class I (AbMito1) abnormal mitochondria.

### 3.2. Reversion of HCV-induced ultrastructural alterations by different antiviral drugs   

It is important to remark that HuH-7 is an immortal line of epithelial-like tumorigenic cells that grow in 2D monolayers. HuH-7.5 was developed from HuH-7. HuH-7.5 is an HCV-replicating cell line because it bears a subgenomic HCV replicon of genotype 2a (JFH-1 strain). These cells contain an HCV minigenome that persistently self-replicates via expression of the viral replicase subunits. The HuH-7.5 cell line is useful because it can efficiently establish HCV replication in cell culture, like a persistent infection. For this study, we renewed the medium as needed according to the pH and maintained the cells until a confluence of 75% was reached. When the cells reached 75% confluence, they were subcultured by centrifugation and then resuspended in fresh medium. The length of culture maintenance depends on the duration of treatment.

**Figure 3:**
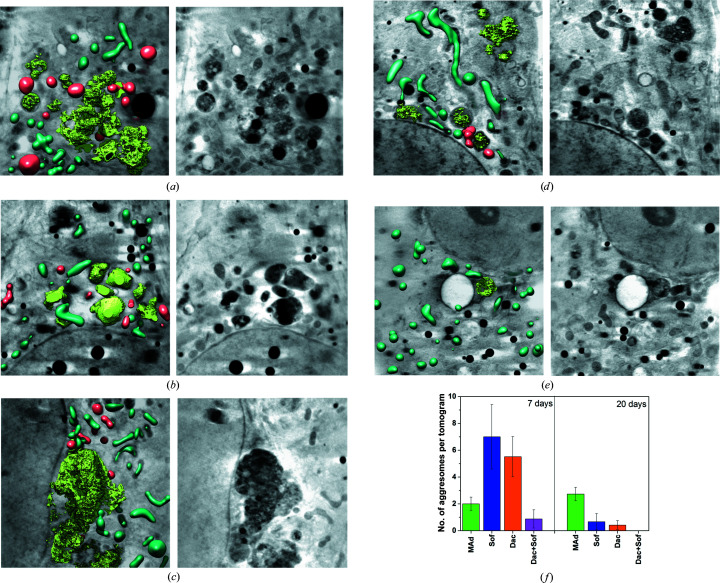
Cryo-SXT of HCV-replicating cells treated with different drugs. (*a*–*e*) Manual segmentation of the surface boundaries identifying the aggresome-like organelles for the different treatments in light green, normal mitochondria in dark green and abnormal mitochondria in red (left column) and volume slices of the tomograms (right column). The scale bar represents 1 µm. (*f*) Statistical analysis of the number of aggresome-like structures in the tomograms per drug-treatment condition.

In order to quantify the reversion of HCV replication, we performed a time course of the healing process by measuring the expression of the viral protein (NS3) in HCV replicon-harbouring cells treated with 2MAd, daclatasvir, sofosbuvir and a combination of sofosbuvir and daclatasvir (Pérez-Berná *et al.*, 2016 ▸). All treatments reduced the viral protein (NS3) expression to levels that were undetectable by Western blotting after 70 h of treatment, showing the efficacy of all of the drugs considered. In order to evaluate the ultrastructural alterations induced by the reversion of HCV replication, we collected tomograms of 15 different cells for each condition after seven and 20 days of treatment. This allowed the identification of a specific cellular structure: a self-repairing organelle that has been found in all treatments and that is segmented in light green in Fig. 3 ▸. This defined organelle is formed by the condensed juxtaposition of membranes similar to the described aggresome (Taylor *et al.*, 2003 ▸; Mishra *et al.*, 2003 ▸; McNaught *et al.*, 2002 ▸; Heath *et al.*, 2001 ▸). This aggresome-like structure may occur in response to cellular stress, probably due to the reversion or degradation of the membranous web during the healing process. Note that around the aggresome-like structure, we found an increased number of lipid droplets and abnormal class I mitochondria (AbMito1; segmented in red in Figs. 3 ▸ *a*–3 ▸ *d*). The healthy mitochondria are segmented in dark green (Figs. 3 ▸ *a*–3 ▸ *e*). As observed in our previous work, during the HCV infection process there is a gradual alteration of the mitochondrial structure concomitant with the extension of the membranous web within the cytoplasm. The spatial localization of abnormal mitochondria surrounding the aggresome-like structure (Fig. 3 ▸) might indicate that the healing process could be localized to specific areas of the cytoplasm and would nucleate and extend from them. We believe that the role of this aggregosome-like structure is in the reversion of the membranous web, and its spatial correlation with the abnormal mitochondria shows an intermediate state of the healing process in the cell (Figs. 3 ▸ *a*–3 ▸ *e*).

In cells treated with 2MAd, dysfunctional mitochondria (AbMito1) are still present in the cytoplasm after seven days of treatment, while these aggresome-like structures can be visualized even after 20 days of treatment (Figs. 3 ▸ *a*, 3 ▸ *b* and 3 ▸ *f*).

After seven days of treatment, the aggresome-like structures were found in higher numbers in cells treated with sofosbuvir (Figs. 3 ▸ *c* and 3 ▸ *f*) than those treated with daclatasvir (Figs. 3 ▸ *d* and 3 ▸ *f*). It is noteworthy that the reduction in number is significant after 20 days of treatment with both sofosbuvir and daclatasvir (Fig. 3 ▸ *f*).

Finally, seven days of treatment with a combination of sofosbuvir and daclatasvir results in cells with an appearance that is virtually indistinguishable from that of the controls, and the aggresome-like structures are visualized as residual small structures in a few cells (Figs. 3 ▸ *e* and 3 ▸ *f*).

#### 3.2.1. PCA analysis of the amide I and lipid regions in the reversion of HCV by direct-acting antivirals   

**Figure 4:**
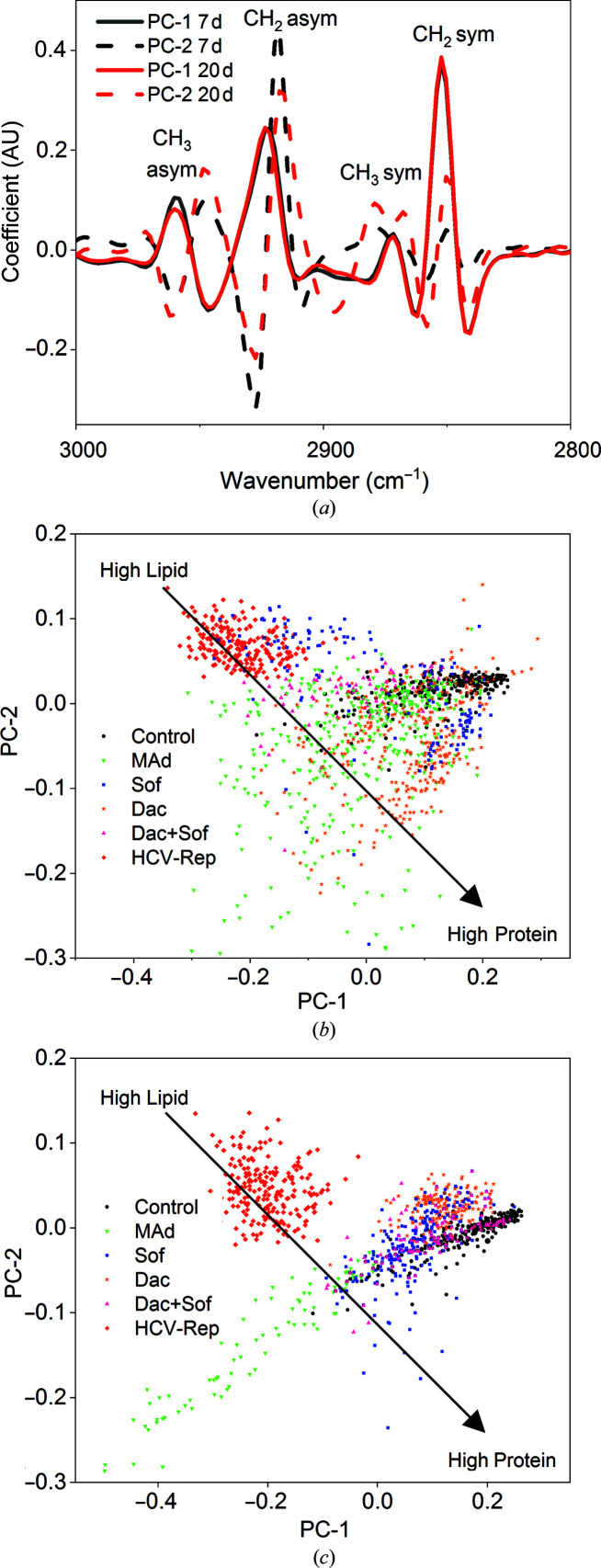
Principal component analysis (PCA) using the second derivative of the lipid region corresponding to control cells (black dots), HCV-replicating cells (red dots) and HCV-replicating cells treated with 2MAd (green dots), daclatasvir (orange dots), sofosbuvir (blue dots) and a combination of daclatasvir and sofosbuvir (pink dots) for seven days (*b*), 20 days (*c*) and loadings (*a*).

The results of PCA are represented in Fig. 4 ▸ in the form of score plots. Each point (score) in the score plot corresponds to one spectrum; thus, each of the six groups (control, HCV-replicating cells and HCV-replicating cells treated with 2MAd, daclatasvir, sofosbuvir and a combination of daclatasvir and sofosbuvir) constitutes 3231 final points that correspond to 15 measured spectra per cell for a total of 30 different cells per condition and for seven and 20 days of treatment, respectively.

In PCA, the loadings are interpreted as the coefficients of the linear combination of the wavenumbers as initial variables from which the principal components are constructed. From a numerical point of view, the loadings are equal to the coordinates of the variables divided by the square root of the eigenvalue associated with the component. In this study, the spectral origins of the variation, which differentiate each data group according to the wavenumbers, are represented in the loadings with positive and negative coefficients correlated to the positive and negative scores, respectively. The score distribution is divided into two principal components, PC1 and PC2. PC1 (accounting for 76% of the total components) is characterized by a maximum in the loadings graph centred at 2850 and 2925 cm^−1^, corresponding to the CH_2_ symmetric and asymmetric stretching absorptions, and a minimum at 2960 cm^−1^ that corresponds to CH_3_ asymmetric stretching, while PC2 (accounting for 16% of the total components) is characterized by a maximum in the loadings graph centred at 2884 and 2947 cm^−1^, corresponding to the CH_2_ asymmetric stretching absorption (Fig. 4 ▸ *a*). Since the PCA was calculated using the second derivative of the spectra, the spectra in the negative part of the PC1 correspond to higher CH_2_ absorption and lower CH_3_ absorption. As described in Benseny-Cases *et al.* (2018 ▸), changes in the lipid:protein absorbance ratio would be reflected in changes in the CH_2_:CH_3_ absorbance ratio as lipids have a much higher CH_2_:CH_3_ ratio than proteins. So, since HCV replication increases the quantity of lipid membranes in the cytoplasm, an increase in CH_2_:CH_3_ is detected in the IR spectra. Taking this into account, the spectra localized on the left part of the PC1 score plot would have a higher lipid:protein ratio (Figs. 4 ▸ *b* and 4 ▸ *c*).

PC2 (accounting for 16% of the total components) also has a minimum in the CH_3_ absorption and a maximum at 2850 cm^−1^ (CH_2_ symmetry), so the spectra now in the positive part of PC2 would have a higher protein content. PC2 also shows a shift of the CH_2_ asymmetric stretching band (centred around 2884, 2947 and 2920 cm^−1^) which highly depends on the lipidic phase environment. Shifts of this group could be a consequence of the lipid:protein ratio but also of changes in the lipid phase (Fig. 4 ▸ *a*).

The score distribution of the two principal components (PC1 and PC2) clearly separates the control (black) and HCV-replicating cell (red) populations (Figs. 4 ▸ *b* and 4*c*). The score plots corresponding to the CH region of the spectrum after seven days and 20 days of treatment are shown in Figs. 4 ▸(*b*) and 4 ▸(*c*), respectively. All treatments after seven days show a large dispersion, indicating high heterogeneity in the reversion process in the cell culture. Indeed, some cells may have already reverted the infection and therefore were located on the positive side, similar to the control cells which were considered to be the reference standard, while others were still reverting the infection and are found in the middle position (Fig. 4 ▸ *b*).

However, after 20 days the DAA treatment groups were located on the control positive side of PC1 (although some dispersion was visible), while the 2MAd-treated group was located in a different cluster still on the negative side, thus implying that HCV replication-related lipid topological alterations were reverted by the DAA drugs considered here to a distribution similar to that of the control cells. Remarkably, the cells treated with 2MAd are clearly differentiated from HCV-replicating cells and control cells in this spectral CH region, probably due to inefficient or/and slower reversion of the membranous web. Note that this group showed a tendency towards the control cluster after 20 days in the relationship between lipids and proteins (Fig. 4 ▸ *c*), although after 20 days we would have expected a similar behaviour to that of the other tested drugs.

#### 3.2.2. Spectral analysis of the lipid, carbonyl and amide I regions   

To better characterize the differences between SR-μFTIR spectra in the lipid, carbonyl and amide I regions, these were vector-normalized and the second derivative was applied. The second derivative allows the observation of small absorption bands and allows the maxima of the peaks (minimums in the second derivative) to be better resolved. The carbonyl peak absorbance and position are important markers of the chemical and oxidative status of the cells and can be used for comparative analysis of the different treatments.

**Figure 5:**
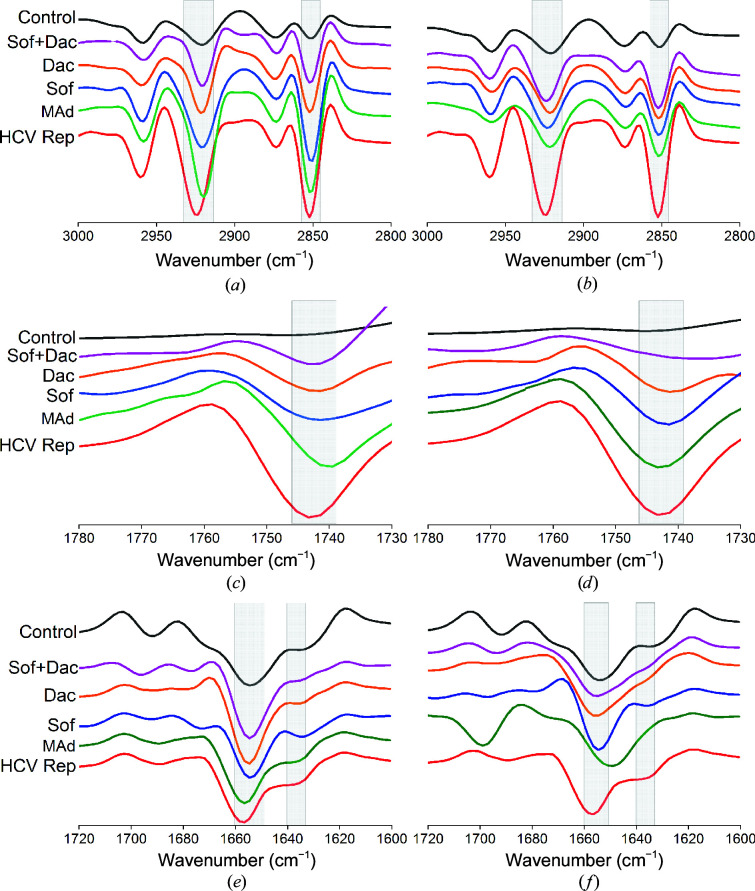
Second derivative of the spectra lipid CH_2_/CH_3_ region (*a*, *d*), carbonyl region (*b*, *e*) and amide I region (*c*, *f*). The black spectrum corresponds to control cells and the red spectrum to HCV-replicating cells. HCV-replicating cells treated with 2MAd are shown as a green spectrum, with daclatasvir as an orange spectrum, with sofosbuvir as a blue spectrum and with a combination of daclatasvir and sofosbuvir as a pink spectrum for seven days (*a*–*c*) and 20 days (*d*–*f*).

In HCV-replicating cells the ER is dilated and long tubular structures of heterogeneous size and vesicles confer a sponge-like cytoplasm. This membranous web proliferation is proportional to an increase in the cellular lipid content in infected cells, as shown in Figs. 1 ▸ and 2 ▸, and the reversion of these alterations should also be reflected in the SR-μFTIR spectra. Indeed, both effects are clearly seen. Initially, the absorption bands at 2925 and 2960 cm^−1^ labelled as light grey areas in Figs. 5 ▸(*a*) and 5 ▸(*d*) correspond to the C—H stretching of the CH_2_ and CH_3_ groups, related to the amount of aliphatic CH groups in lipids and amino acids in the proteins, respectively. The SR-μFTIR data at these frequencies showed firstly an increase of the peak intensity in the HCV-replicating cells with respect to the control cells and secondly a decrease in the absorption peaks for all treated cells due to the effect of the drugs and the subsequent reversion of the membranous web. Moreover, the effectivity of the drugs in the reversion of the membranous web is proportional to the decrease of the absorption at 2925 and 2960 cm^−1^.

As described previously, in HCV-replicating cells the mitochondria displayed significant alterations such as matrix condensation and dilated cristae (Pérez-Berná *et al.*, 2016 ▸). Also, the modified ER tubules form a complex network surrounding the abnormal mitochondria. Since dysfunctional mitochondria are a major source of reactive oxygen species (ROS; Ježek *et al.*, 2018 ▸), all of these rearrangements in the infected cell may change the oxidation state of the cell. This notion is supported by the SR-μFTIR spectra, with a change in the intensity of the band at 1740 cm^−1^ as an indicator of lipid oxidation in the cells (Figs. 5 ▸ *b* and 5 ▸ *e*). The strongest second-derivative absorbance at 1740 cm^−1^ is found in the HCV-replicating cell samples (Figs. 5 ▸ *b* and 5 ▸ *e*, red curve), while the weakest carbonyl absorbance was found in the control cells (Figs. 5 ▸ *b* and 5 ▸ *e*, black curve), highlighted with a light grey area in Figs. 5 ▸(*b*) and 5 ▸(*e*). HCV-replicating cells treated for seven days with a combination of sofosbuvir and daclatasvir (Fig. 5 ▸ *b*, pink curve) showed the lowest oxidation of all treatments, and after 20 days of treatment (Fig. 5 ▸ *e*, pink curve) the carbonyl absorbance was similar to that of the control cells. In contrast, the carbonyl absorbances in cells treated with 2MAd for seven and 20 days were comparable with the spectra of infected cells (Figs. 5 ▸ *b* and 5 ▸ *e*, green curve). Therefore, the intensity of the carbonyl peak decreases with the degree of reversion of mitochondrial alterations observed after DAA treatment.

An evaluation of the amide I region spectra, which roughly indicate possible changes in protein conformation and structure, was performed to identify specific changes in the different conditions (Figs. 5 ▸ *c* and 5 ▸ *f*). The infrared spectrum of the amide I′ region is formed by different underlying components that give rise to a broad and asymmetric band with a maximum at about 1655 cm^−1^. This has been associated with a dominant α-helical secondary structure (Goormaghtigh *et al.*, 1990 ▸), as seen here for all conditions, although the peak position and intensity slightly varies depending on the sample group (Figs. 5 ▸ *c* and 5 ▸ *f*).

Remarkably, after 20 days of treatment with a combination of sofosbuvir and daclatasvir the percentage of secondary structures was analogous to the control cells (Fig. 5 ▸ *f*, pink curve), probably due to the presence of proteins that make the healing process possible. Note that in the case of 2MAd (Fig. 5 ▸ *f*, green curve) after 20 days of treatment the amide I′ region shows an apparent peak at 1695 cm^−1^ that together with the band at 1630 cm^−1^ could correspond to a nonfibrillar intermolecular β-sheet (Dong *et al.*, 1997 ▸). The increase in this secondary structure, which is typically present in refolding protein precipitates (Pérez-Berná *et al.*, 2008 ▸), could also be related to the presence of aggresome-like structures in the cells. These two events are clearly a consequence of the ongoing reversion of the viral structures that form the membranous web.

#### 3.2.3. Analysis of the ν(CH_2_:CH_3_), ν(CH_2_:C=O) and ν(C=O:CH) ratios   

**Figure 6:**
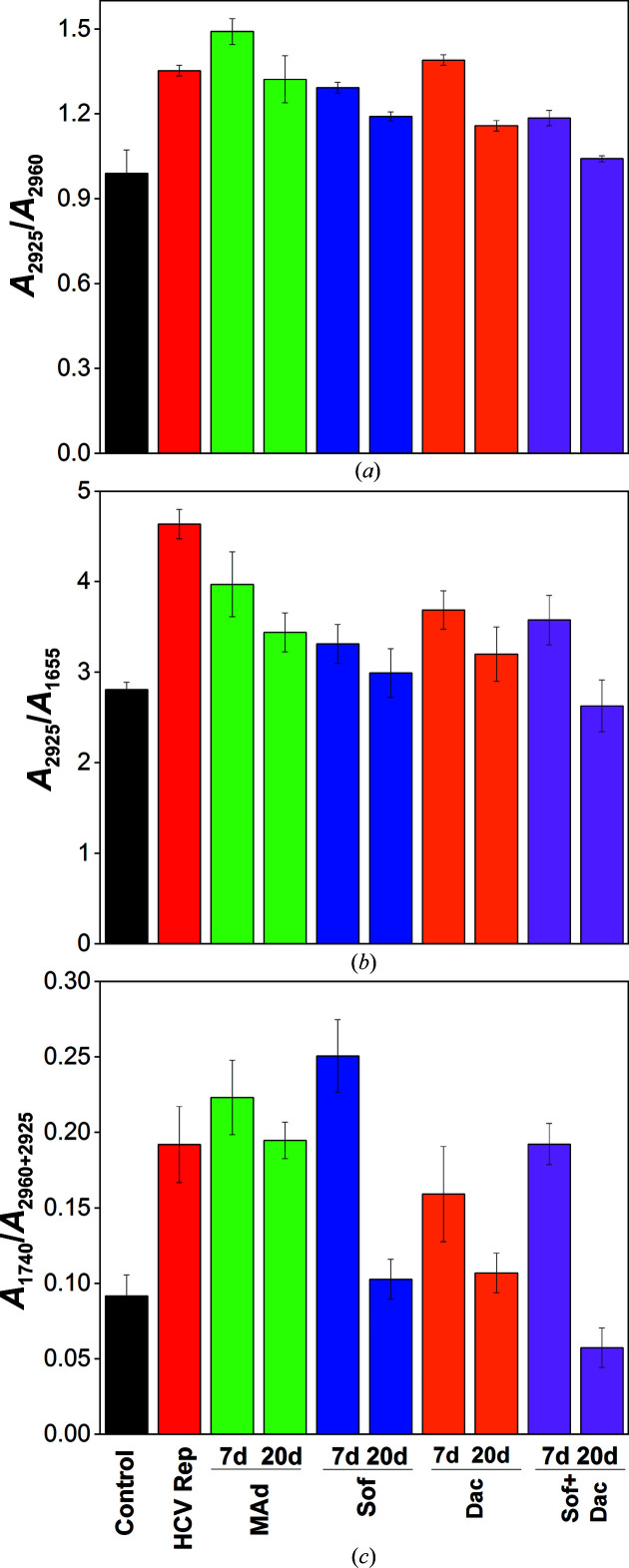
Statistical analysis of the spectral changes corresponding to changes in the alkyl:methyl ν(CH_2_:CH_3_) ratio (*A*_2925_/*A*_2960_) (*a*), lipid:protein ratio (*A*_2925_/*A*_1655_) (*b*) and lipid carbonyl:CH_2_+CH_3_ ratio (*c*), expressed as the ratio of the corresponding absorbance values, for seven and 20 days of treatment. The ratios were calculated using all spectra in each segment. Standard errors of the mean (SEM) were calculated. Black columns correspond to control cells and red columns to HCV-replicating cells. HCV-replicating cells treated with 2MAd are shown as green columns, with daclatasvir as orange columns, with sofosbuvir as blue columns and with a combination of daclatasvir and sofosbuvir as pink columns.

The CH_2_:CH_3_ absorbance ratio might be considered as a marker of cell growth or a reflection of the lipid:protein absorbance ratio (Gasparri & Muzio, 2003 ▸). Therefore, we have processed the IR absorbance intensities in the region of asymmetrical stretching of CH_2_ and CH_3_ groups (2925 and 2960 cm^−1^, respectively), defining the positions of the respective bands for each sample condition to evaluate this ratio. We found an increase in the CH_2_:CH_3_ ratio in infected cells, which could be related to the formation of the membranous web (Fig. 6 ▸ *a*, red bar). The measurement of the *A*_2925_/*A*_1655_ ratio, corresponding to the lipid:protein ratio (Fig. 6 ▸ *b*), shows a very similar pattern to the *A*_2925_/*A*_2960_ ratio and further supports our interpretation of changes in the *A*_2925_/*A*_1655_ ratio as changes in the lipid:protein ratio. As shown by Benseny-Cases *et al.* (2018 ▸), the increase in the lipid:protein ratio linearly correlates with the *A*_2925_/*A*_2960_ ratio.

HCV replicon-bearing cells treated with the various drugs showed a statistical increase in the *A*_2925_/*A*_2960_ absorbance ratio after seven days of treatment (Fig. 6 ▸ *a*). However, the CH_2_:CH_3_ absorbance ratio decreased in all of the drug treatments after 20 days without an appreciation in apoptotic cells in the SXT-evaluated grids. Therefore, this general reduction in the ratio after 20 days of treatment could be related to a diminution of the viral structures that form the membranous web and would imply a reduction in the total lipid cellular content during reversion of the infection (Fig. 6 ▸ *a*). As a control, human hepatoma HuH-7 cells were also treated with each of the drugs and no statistical changes in the ratios were found after 20 days of treatment. For this reason, the differences in the healing process with the different drugs could be ascribed to differences in the kinetics or the nature of the processes underlying clearance of the viral structures in the HCV replicon-bearing cells.

Analysis of the *A*_2925_/*A*_1655_ ratio confirms that the HCV-replicating cells showed a higher lipid:protein ratio compared with the control cells, probably due to the formation of the membranous web (Fig. 6 ▸ *b*, red bar). Remarkably, a decrease in the ratio is shown for all treated conditions; similar ratio values to the control cells were recovered after 20 days. This may reflect the reduction of the membranous web and the healing of the cells (Fig. 6 ▸ *b*).

**Figure 7:**
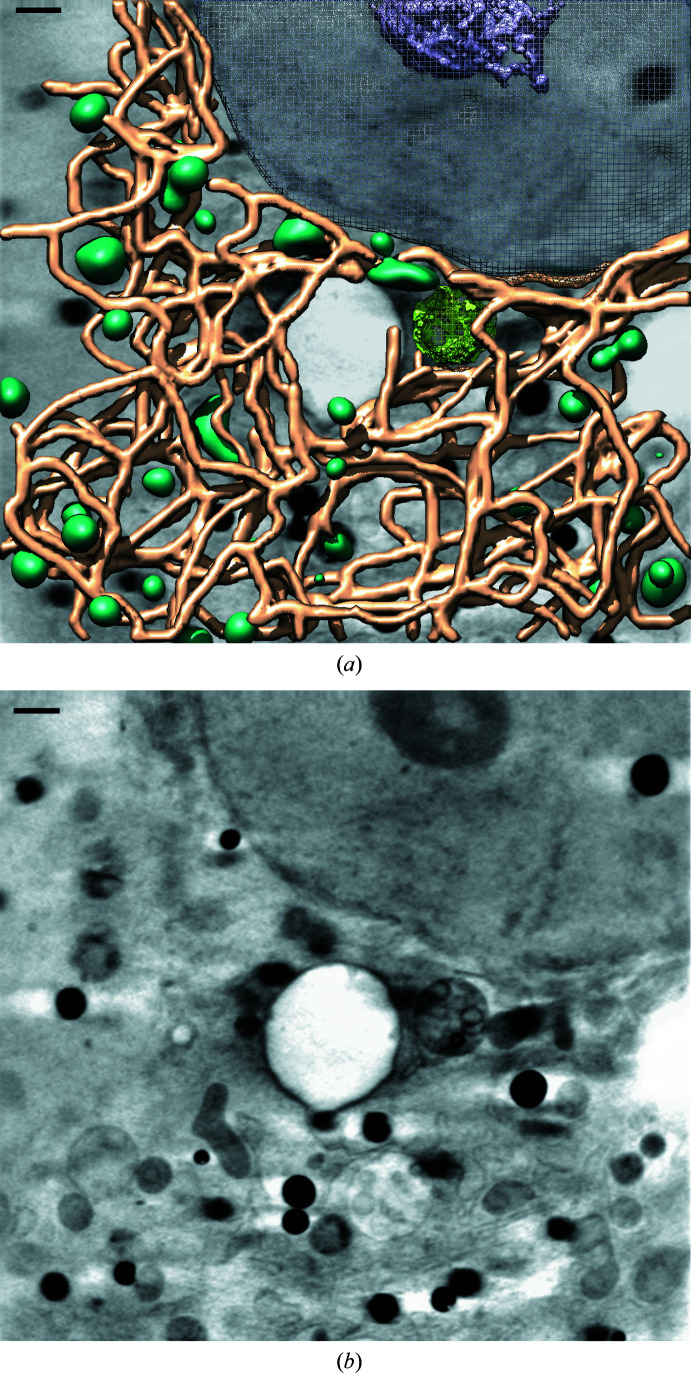
Cryo-SXT of HCV-replicating cells treated with DAAs. (*a*) Manual segmentation of the surface boundaries identifying the organelles: aggresome-like structure in light green, normal mitochondria in dark green, ER in brown, nuclear envelopment in mesh and nucleolus in purple. (*b*) Volume slices of the tomograms. The scale bar represents 1 µm.

Finally, in order to study the sample oxidation state, we looked at the intensity of the band at 1740 cm^−1^ as an indicator of lipid oxidation and referred it to the sum of the intensities of the CH absorption at 2960 and 2925 cm^−1^ (Benseny-Cases *et al.*, 2018 ▸). Fig. 6 ▸(*c*) represents the *A*_1740_/*A*_2925+2960_ ratio defining the oxidation state of the sample. It is notewothy that the HCV-replicating cells showed a ratio which was double that of the control cells, whereas a significant increase in the ratio is observed after seven days of treatment with 2MAd or sofosbuvir, but not for treatment with daclastavir or a combination of sofosbuvir and daclastavir (Fig. 6 ▸ *c*). This can be interpreted as an indication of oxidation due to phospholipid alkyl-chain cleavage and subsequent aldehyde-group formation, which could be related to turnover of the cellular lipidic pathway during reversion of the infection and modification of the altered cytoplasm (Fig. 6 ▸ *c*). In contrast, after 20 days of treatment with all drugs except 2MAd the *A*_1740_/*A*_2925+2960_ ratio showed a complete reduction, bringing the value of the ratio to that of the control or even lower, such as for the combination of sofosbuvir and daclatasvir. This may reflect not only complete reversion of the membranous web, as shown in Fig. 7 ▸, but also that the cellular structure and the oxidation state of the lipid in the cells is recovered to the level in the control cells.

Finally, treatment with a combination of sofosbuvir and daclatasvir results in cells with an appearance that is virtually indistinguishable from that of the control cells, with virtually no membranous network; the mitochondria displayed a normal appearance and there was no increase in the presence of cytoskeleton fibres (Fig. 7 ▸). Treatment with the optimal antiviral drug combination sofosbuvir and daclatasvir reverses HCV replication, viral protein expression and the oxidation state of the lipid in the cells, with virtually complete reversion to the control cells.

## 4. Conclusions   

By combining cryo-SXT and SR-μFTIR correlatively on the same samples, we have linked an overall increase in the oxidation of the cytoplasm of the HCV-replicating cell with its profound structural alteration. In addition, we have identified the chemical hallmarks of the infection which are reverted during the healing process. Also, specialized aggresome-like structures that are only present in treated cells could be related to cellular recovery after HCV infection. The correlation of both techniques allows a better understanding of the complexity of the system linking general composition changes to structural changes.

The specific spectral differences found between control and HCV-replicating cells are related to changes in protein and lipid distribution (Fig. 2 ▸). As no significant structural change is found in the nucleus during HCV infection, the spectral changes observed by SR-μFTIR should be solely related to the formation of the membranous web and the concomitant mitochondrial dysfunction. This 3D morphological reorganization was also chemically visible in the SR-μFTIR 2D maps, with peaks of lipid distribution in the perinuclear region (Figs. 2 ▸ *e* and 2 ▸ *f*); also, the maximum protein distribution (Figs. 2 ▸ *e* and 2 ▸ *g*) was localized in the region where the mitochondria presented hallmarks of morphological dysfunction, probably due to the increase in viral proteins during the formation of the tubular network for viral replication. Besides, these areas also presented high oxidation parameters and abnormal mitochondria surrounded by the membranous web, which are both probably due to the strong viral ER transformation into the replicating viral factories located inside the membranous web.

There are different existing treatments against HCV infection that provide different effectivities in reversion of the infection. In this study, we have selected one widely used experimental inhibitor in cell culture, 2MAd, and clinically relevant drugs such as sofosbuvir (an inhibitor of NS5B polymerase) and daclatasvir (an NS5A inhibitor), and a combination of both. Here, we have focused on evaluating the reversion of HCV infection due to the different drug treatments by SR-μFTIR. With regard to the PCA scores of the lipid spectral regions (CH stretching and scissoring) the control and replicating cells were clearly distinct (Fig. 4 ▸). The increase in lipid and protein content is related to the development of the membranous web. The reversion of the infection triggered by all treatments after seven days still shows high dispersion in the PCA score. The inhibition of RNA viral replication by daclatasvir exhibits a PCA score compatible with a healing process mediated by a decrease in the protein and lipid content in HCV-replicating cells. This effect could be due to an abrupt retreat of the membranous web (Fig. 4 ▸). A more gradual effect was visible for sofosbuvir, which only inhibits the NS5B polymerase and showed a slight decrease in the lipid:protein ratio (Fig. 4 ▸). The combination of sofosbuvir and daclatasvir showed the largest cell population that reverted the protein and lipid content to levels of the control population after 20 days of treatment (Fig. 4 ▸).

Another important SR-μFTIR feature regarding lipids is the carbonyl group stretching vibration, generating a band near 1740 cm^−1^ that is related to oxidation stress. This band peak and the indicative *A*_1740_/*A*_2925+2960_ ratio observed in the control cells were both very low (Figs. 5 ▸ *b*, 5 ▸ *e* and 6 ▸ *c*), while in replicating cells the carbonyl band peak was clearly visible and the *A*_1740_/*A*_2925+2960_ ratio was almost double that of the control cells (Figs. 5 ▸ *b*, 5 ▸ *e* and 6 ▸ *c*). This can be interpreted as an indication of cellular oxidation stress due to phospholipid alkyl-chain cleavage and subsequent aldehyde-group formation due to the development of the membranous web. One interesting finding is the decrease in the carbonyl band in the HCV-replicating cells treated overall with a combination of daclatasvir and sofosbuvir (Figs. 5 ▸ *b*, 5 ▸ *e* and 6 ▸ *c*). Taking all of the results into account, the most efficient DAA treatment for the reversion of HCV replication is the combination of daclatasvir and sofosbuvir after both seven and 20 days, as shown in Fig. 7 ▸.

Nevertheless, HCV replicon-bearing cells treated with 2MAd for 20 days showed a cell population that presented a reduction in protein and lipid content, with a high oxidation and high lipid:protein ratio. Previously (Pérez-Berná *et al.*, 2016 ▸), we have shown that after three days of treatment viral RNA or NS5 protein were not detected by Western blotting. However, the cells presented important post-infection structural alterations such as aggresome-like structures, abnormal mitochondria and prominent cytoskeleton fibres (Pérez-Berná *et al.*, 2016 ▸), features that have been chemically related to the SR-μFTIR results here. Finally, after 20 days with 2MAd there is a clear healing tendency toward the control cell state, although this treatment is less effective or much slower than the clinically approved direct antivirals as alterations are still present in the healing cells.

Analysis of the amide I′ region allowed evaluation of the secondary structures present in the protein. It showed a slight tendency towards β-sheet secondary structures associated with HCV infection. This is explained by the fact that HCV proteins have a compact, globular domain structure, consisting mostly of β-strands and random coil with α-helices (Penin *et al.*, 2004 ▸).

For all treatments, the increase in protein aggregation, the high lipid:protein ratio and the cellular oxidation stress could be related to the presence of large self-repairing structures such as the aggresome organelle (Taylor *et al.*, 2003 ▸; Mishra *et al.*, 2003 ▸; McNaught *et al.*, 2002 ▸; Heath *et al.*, 2001 ▸). These structures were also present, although to a lesser extent, in the seven-day DAA treatments. The formation of the aggresome-like structure may be an important consequence of the membranous web degradation. The process for membranous web regression may imply the formation of these specialized organelles.

Finally, the correlation between cryo soft X-ray tomography and SR-μFTIR microscopy allows a relationship to be set up between the membranous web in the cells and the specific chemical footprint of lipids and proteins. As shown here, the reversion of the infection due to specific drug treatments and the possible side effects that these treatments might have could be followed by PCA of the CH region. This correlated study between the MISTRAL and MIRAS beamlines could clarify the chemical nature of the viral structures during infection and also during the healing process. Our results also constitute a proof of concept for the use of cryo-SXT as a platform that enables determination of the potential impact of candidate compounds on the cell ultrastructure, which may assist in drug development at a preclinical level.

## 5. Data availability   

The original imaging data referenced in the manuscript have been deposited in EMPIAR (https://www.ebi.ac.uk/pdbe/emdb/empiar/) with accession code EMPIAR-10789.
